# P-588. Risk Perception and Communication about Lyme Disease Prevention by Healthcare Providers: A Mixed-Methods Study

**DOI:** 10.1093/ofid/ofaf695.802

**Published:** 2026-01-11

**Authors:** L Hannah Gould, Stephanie A Duench, James H Stark, Niyati Patel, Matt Killingsworth, Jason Riis

**Affiliations:** Pfizer Vaccines, New York, New York; Pfizer, Collegeville, Pennsylvania; Pfizer Biopharma Group, Collegeville, Pennsylvania; Behavioralize, WYNNEWOOD, PA; Behavioralize, WYNNEWOOD, PA; Behavioralize, WYNNEWOOD, PA

## Abstract

**Background:**

Nearly 500,000 individuals are diagnosed with Lyme disease (LD) annually in the U.S. As healthcare providers (HCPs) play an essential role in supporting patients to make prevention decisions for LD and other tickborne diseases, it is critical to understand how they (a) decide whether to initiate discussions on tick-bite prevention, (b) frame those discussions for patients, and (c) what beliefs drive these decisions.

**Methods:**

We conducted a mixed-methods study among HCPs practicing in high LD incidence states. First, we conducted in-depth qualitative interviews (n = 10, 60 minutes each) to explore decision-making and communication strategies. Findings from the interviews informed a subsequent online survey (n = 124) to quantify the prevalence of key behaviors and attitudes. Survey topics included practice context, personal protective measures conversations, and perceived risk.

**Results:**

Most (83%) HCPs identified tick bite prevention as a moderate, high, or top priority; however, prevention was discussed in only 24% of annual wellness visits, primarily due to time constraints and competing priorities. When discussed, HCPs reported spending an average of 3.5 minutes on the topic.

Most (73%) prevention discussions occurred after patients asked about ticks/LD or mentioned something to prompt the conversation. Only 19% of HCPs proactively included it in their agenda. Proactive HCPs reported nearly 4 times as many prevention discussions, addressing the topic in 57% of wellness visits compared to just 16% among non-proactive HCPs.

Perceptions of patient risk varied by practice setting. Providers in rural areas reported the highest proportion of at-risk patients, followed by those in small towns, suburban areas, and urban centers.

**Conclusion:**

Most HCPs take a reactive approach to tick-bite prevention, missing opportunities to educate patients. The difference in discussion frequency between proactive and non-proactive HCPs is consistent with the possibility that the inclusion of tick-bite prevention in wellness visit agendas could increase prevention conversations. Future research should explore strategies for efficient, proactive prevention discussions in scheduled appointments.

**Disclosures:**

L. Hannah Gould, PhD, MS, MBA, Pfizer Inc.: Employee; may hold company shares and/or stocks. Stephanie A. Duench, PhD, Pfizer Vaccines: Stocks/Bonds (Public Company) James H. Stark, PhD, Pfizer Vaccines: Employer|Pfizer Vaccines: Stocks/Bonds (Public Company) Niyati Patel, MS, Pfizer: Advisor/Consultant Matt Killingsworth, PhD, Pfizer: Advisor/Consultant Jason Riis, PhD, Pfizer: Advisor/Consultant

